# Genome-Wide Evolutionary Analyses of G1P[8] Strains Isolated Before and After Rotavirus Vaccine Introduction

**DOI:** 10.1093/gbe/evv157

**Published:** 2015-08-08

**Authors:** Mark Zeller, Celeste Donato, Nídia Sequeira Trovão, Daniel Cowley, Elisabeth Heylen, Nicole C. Donker, John K. McAllen, Asmik Akopov, Ewen F. Kirkness, Philippe Lemey, Marc Van Ranst, Jelle Matthijnssens, Carl D. Kirkwood

**Affiliations:** ^1^Laboratory of Clinical Virology, University of Leuven, Leuven, Belgium; ^2^Enteric Virus Research Group, Murdoch Childrens Research Institute, Royal Children's Hospital, Parkville, VIC, Australia; ^3^Department of Microbiology, La Trobe University, Bundoora, VIC, Australia; ^4^Laboratory Evolutionary and Computational Virology, University of Leuven, Leuven, Belgium; ^5^The J. Craig Venter Institute, Rockville, MD

**Keywords:** rotavirus, G1P[8], vaccine introduction, phylodynamics

## Abstract

Rotaviruses are the most important etiological agent of acute gastroenteritis in young children worldwide. Among the first countries to introduce rotavirus vaccines into their national immunization programs were Belgium (November 2006) and Australia (July 2007). Surveillance programs in Belgium (since 1999) and Australia (since 1989) offer the opportunity to perform a detailed comparison of rotavirus strains circulating pre- and postvaccine introduction. G1P[8] rotaviruses are the most prominent genotype in humans, and a total of 157 G1P[8] rotaviruses isolated between 1999 and 2011 were selected from Belgium and Australia and their complete genomes were sequenced. Phylogenetic analysis showed evidence of frequent reassortment among Belgian and Australian G1P[8] rotaviruses. Although many different phylogenetic subclusters were present before and after vaccine introduction, some unique clusters were only identified after vaccine introduction, which could be due to natural fluctuation or the first signs of vaccine-driven evolution. The times to the most recent common ancestors for the Belgian and Australian G1P[8] rotaviruses ranged from 1846 to 1955 depending on the gene segment, with VP7 and NSP4 resulting in the most recent estimates. We found no evidence that rotavirus population size was affected after vaccine introduction and only six amino acid sites in VP2, VP3, VP7, and NSP1 were identified to be under positive selective pressure. Continued surveillance of G1P[8] strains is needed to determine long-term effects of vaccine introductions, particularly now rotavirus vaccines are implemented in the national immunization programs of an increasing number of countries worldwide.

## Introduction

Rotavirus is the most common viral cause of acute gastroenteritis in infants and young children worldwide. Rotavirus infection results in 114 million episodes of gastroenteritis, 24 million clinic visits, and 2.4 million hospitalizations annually (Glass et al. 2006). Of the estimated 453,000 annual deaths, the majority occur in developing nations in Asia and sub-Saharan Africa (Tate et al. 2012). Rotavirus belongs to the *Reoviridae* virus family and has a double-stranded RNA genome composed of 11 gene segments. The genome encodes six structural viral proteins (VP1−4, VP6, VP7) and six nonstructural proteins (NSP1−5/6) (Estes and Greenberg 2013). Numerous mechanisms impact the dynamics of rotavirus diversity including genetic shift, genetic drift, recombination, and zoonotic transmission. The accumulation of spontaneous sequential point mutations (genetic drift) occurs due to the error-prone nature of the rotavirus RNA-dependent RNA polymerase (Estes and Greenberg 2013). The rate of mutations has been calculated for several VP7 genotypes, and a small number of other genes including VP4 and NSP2, resulting in the identification of varying mutation rates that may reflect the different selective pressures exerted on different genes and genotypes (Jenkins et al. 2002; Matthijnssens, Heylen, et al. 2010; Donker and Kirkwood 2012; Nagaoka et al. 2012; Trang et al. 2012).

Rotaviruses are classified into eight groups or species (A−H), with group A rotavirus strains being the most common cause of disease in humans (Matthijnssens et al. 2012). A full genome genotyping classification system for group A rotaviruses based on the open reading frame (ORF) of each gene has been established: Gx-P[x]-Ix-Rx-Cx-Mx-Ax-Nx-Tx-Ex-Hx (Matthijnssens et al. 2011). To date, 27 G (VP7), 37 P (VP4), 17 I (VP6), 9 R (VP1), 9 C (VP2), 8 M (VP3), 18 A (NSP1), 10 N (NSP2), 12 T (NSP3), 15 E (NSP4), and 11 H (NSP5) genotypes have been described (Matthijnssens et al. 2011; Guo et al. 2012; Papp et al. 2012; Trojnar et al. 2013; Jere et al. 2014). This extends the classic classification system based on the two outer capsid proteins into G (glycoprotein, VP7) and P (protease sensitive, VP4) genotypes, respectively (Estes and Greenberg 2013). G1P[8] is the dominant genotype in countries across the globe and typically exhibits the archetypal constellation G1-P[8]-I1-R1-C1-M1-A1-N1-T1-E1-H1) (Santos and Hoshino 2005; Matthijnssens and Van Ranst 2012). In the prevaccine period, surveillance data from Belgium and Australia indicated that G1P[8] was the dominant genotype. However, rotavirus genotype distributions fluctuated both geographically and temporally in the absence of vaccination (Kirkwood 2010; Zeller et al. 2010).

Two live-oral vaccines are currently available on the global market, Rotarix (GlaxoSmithKline Vaccines, Belgium) and RotaTeq (Merck and Co., USA), and included in the routine vaccination programs of many countries including the United States, Brazil, Belgium, and Australia (Dennehy 2012). RotaTeq is a live-attenuated pentavalent vaccine that contains five genetically distinct human–bovine reassortant virus strains. Each reassortant strain contains a human rotavirus gene encoding one of the outer capsid proteins (VP7 encoding G1, G2, G3, or G4; or VP4 encoding P[8]) within a bovine WC3 strain backbone (G6P[5]) (Heaton et al. 2005; Matthijnssens, Joelsson, et al. 2010). RotaTeq is administered in a three dose schedule at 2, 4, and 6 months of age. Rotarix is a live-attenuated monovalent vaccine composed of a G1P[8] strain that is administered in a two dose schedule at 2 and 4 months of age (Vesikari et al. 2007).

In early 2006, Rotarix and RotaTeq became commercially available in Australia and were subsequently introduced into the Australian National Immunisation Program in July 2007. Each state and territory independently selected which vaccine to implement; Victoria, Queensland, Western Australia, and South Australia used RotaTeq, while New South Wales, the Northern Territory, Tasmania, and the Australian Capital Territory used Rotarix (Buttery et al. 2011). By December 2008, the estimated national vaccine coverage was 87% for at least one dose of vaccine received by 4 months of age, and 84% for a full vaccine course (either two or three doses) by 13 months of age (Macartney and Burgess 2009). Belgium was the first country in the European Union to introduce rotavirus vaccines into the routine childhood immunization schedule with Rotarix and RotaTeq introduced in November 2006 and June 2007, respectively (Braeckman et al. 2011). Vaccine coverage reached approximately 90% by the start of the 2007–2008 rotavirus season and the most frequently used vaccine in Belgium is Rotarix (Zeller et al. 2010; Braeckman et al. 2011). Several studies have shown that vaccine introduction has significantly decreased the burden of rotavirus disease in Australia and Belgium (Lambert et al. 2009; Zeller et al. 2010; Braeckman et al. 2011; Buttery et al. 2011). It is predicted that vaccine introduction will result in an increase in selective pressure exerted on wild-type rotavirus strains in the population, affecting the evolution of these strains.

The aim of this study was to genetically characterize circulating G1P[8] strains in Belgium (Rotarix) and Melbourne, Australia (RotaTeq), and to investigate viral evolution in the pre- and postvaccine era.

## Materials and Methods

### Sample Preparation

Stool samples were collected from children hospitalized under the age of five as part of the ongoing rotavirus surveillance studies in Belgium and Australia. Belgian samples were collected from the Gasthuisberg University Hospital, Leuven, and from other hospitals in Belgium between 1999 and 2010, and G1P[8] rotaviruses were selected based on the phylogenetic diversity of VP7 and availability of stool sample. Australian stool samples were collected from the Royal Children’s Hospital, Melbourne, Australia, between 2001 and 2011 and were randomly selected based on the availability of stool samples and the proportion of G1P[8] samples collected in a given year. No Group A rotavirus strains were included that were completely or partially vaccine derived.

For each stool sample, RNA extractions were performed using the QIAamp viral RNA mini kit (Qiagen, Hilden, Germany) and these were sent to the J. Craig Venter Institute (Rockville, MD) for sequencing as previously described (McDonald et al. 2009, 2012). The sequences generated in this study were deposited in GenBank under the accession numbers listed in supplementary table S1, Supplementary Material online.

### Bayesian Evolutionary Inference and Phylogenetic Network Analysis

We estimated time-measured evolutionary histories for each rotavirus segment using Bayesian phylogenetic inference as implemented in BEAST (Drummond et al. 2012). The nucleotide substitution process was modeled by separately partitioning the codon positions into 1st + 2nd and 3rd positions (Shapiro et al. 2006) and applying a separate general time-reversible substitution model with gamma-distributed rate heterogeneity and a proportion of invariant sites (GTR + I + gamma) (Tavaré 1986), under an uncorrelated lognormal relaxed molecular clock to account for variation in rates of evolution among lineages (Drummond et al. 2006). We specified a Bayesian Skygrid coalescent tree prior that allows the population size to be estimated through time from a single or multiple unlinked genetic loci (Gill et al. 2013).

Three independent Markov chain Monte Carlo chains were run for 100 million steps and sampled every 10,000th generation, with 10% of the generations discarded as chain burn-in. All analyses were performed using the BEAGLE library to enhance computation speed (Suchard and Rambaut 2009; Ayres et al. 2012). Convergence and mixing of the chains were inspected using Tracer version 1.5; all continuous parameters yielded effective sample sizes greater than 200. A maximum clade credibility tree was summarized using TreeAnnotator version 1.7.4 (Drummond and Rambaut 2007). In addition, a phylogenetic network was constructed of all 11 concatenated ORFs using the SplitsTree4 program (Huson and Bryant 2006). Based on the phylogenetic network, clusters were defined and associations among clusters, country of isolation, and period of sample collection (before or after vaccine introduction) were determined using Fisher’s exact test.

### Selection Pressure Analysis

To estimate site-specific nonsynonymous/synonymous substitution rate ratios (*d*_N_/*d*_S_), we used the Renaissance counting methodology that combines estimates of the number of synonymous and nonsynonymous substitutions at each site with an empirical Bayes procedure to produce a posterior distribution of *d*_N_/*d*_S_ ratios for all sites in the alignment (Lemey et al. 2012). In addition, *d*_N_/*d*_S_ ratios were also computed using the Mixed Effects Model of Evolution (MEME), Single likelihood ancestor counting (SLAC), Fixed Effects Likelihood (FEL), and Fast Unconstrained Bayesian AppRoximation (FUBAR) algorithms as implemented in the Datamonkey webserver (Pond et al. 2005; Delport et al. 2010). As outcomes can differ considerably depending on the chosen method, we only considered sites that were under positive selection pressure according to three or more of the five abovementioned methods.

## Results

**Fig. 1.—:**
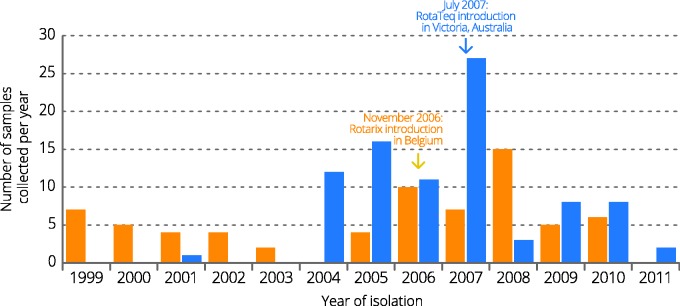
Sampling distribution of G1P[8] rotaviruses used in this study. Australian G1P[8] strains are indicated in blue, whereas Belgian G1P[8] strains are indicated in orange. For each year, the number of selected strains is shown.

The complete ORF of all 11 gene segments of 69 and 88 G1P[8] rotaviruses from Belgium and Australia, respectively, isolated before and after vaccine introduction were determined. In total, 78 G1P[8] strains were detected before vaccine introduction and 79 strains were detected after vaccine introduction. The majority of the Australian samples were collected after 2004, whereas Belgian samples were collected from 1999 until 2010 although no samples were collected in 2004 (fig. 1).

**Fig. 2.—:**
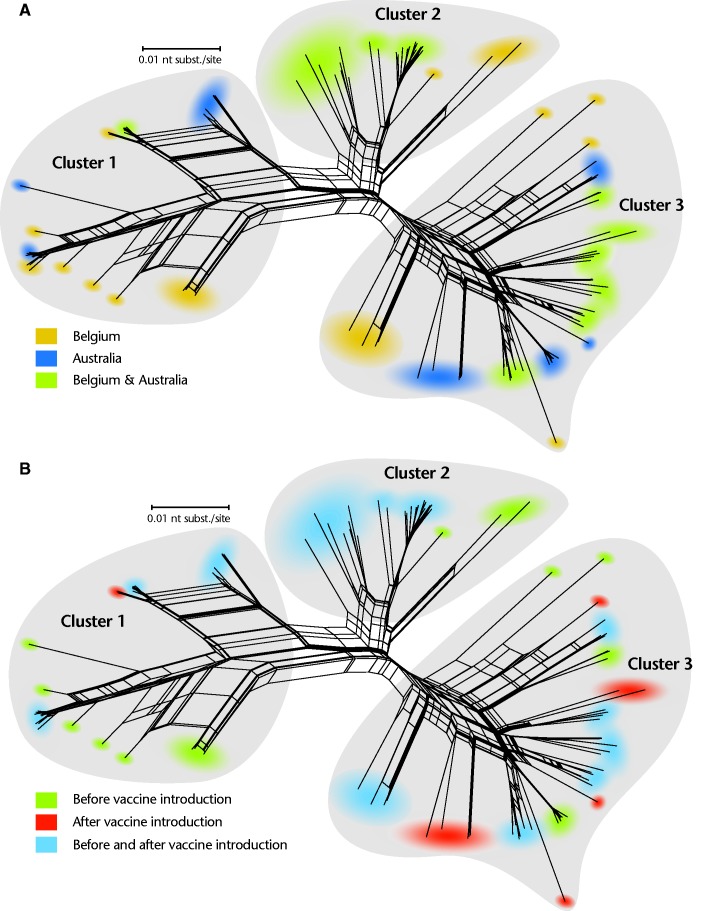
Phylogenetic network of 69 Belgian and 88 Australian concatenated G1P[8] strains. Branches are drawn to scale and splits in the network indicate reassortments. Clusters are color coded according to the legend by country (*A*) or by isolation before or after vaccine introduction (*B*).

The level of reassortment in the ancestral history of the Belgian and Australian G1P[8] rotaviruses was investigated by constructing a phylogenetic network from concatenated sequences of the 11 genes (fig. 2*A*). The phylogenetic network showed that the G1P[8] strains were distributed among three main clusters, each containing multiple subclusters. The presence of a large number of incompatible splits in the network indicates the occurrence of frequent reassortment events between strains belonging to the same or different clusters. Belgian and Australian G1P[8] strains were found in each of the three clusters, although only six Australian strains were observed in cluster 2. Within every cluster, separate subclusters could be identified containing only strains from either Belgium or Australia. However, several subclusters (shaded green in fig. 2*B*), predominately in cluster 2 and 3, contained both Belgian and Australian strains reflecting rapid global dissemination of G1P[8] rotaviruses. No overall association was observed between the country of isolation and the period of sampling (pre- or postvaccine introduction) (table 1). Focusing on specific clusters, the country of isolation and the period of sampling were not significantly related to each other for cluster 2 and 3 (*P* = 0.37 and *P* = 0.33, respectively). For cluster 1, however, less strains were isolated in Belgium after rotavirus vaccine introduction (*P* = 0.01).

**Table 1:** Distribution of Samples Per Cluster, Country of Collection, and Date of Collection

	Number of Samples Isolated in the Prevaccine Introduction Period (%)	Number of Samples Isolated in the Postvaccine Introduction Period (%)	Total
Overall			
Australia	42 (52.3)	46 (47.7)	88
Belgium	36 (47.8)	33 (52.2)	69
Total	79 (50.3)	78 (49.7)	157
Cluster I			
Australia	15 (41.7)	21 (58.3)	36
Belgium	13 (81.3)	3 (18.8)	16
Cluster 2			
Australia	1 (16.7)	5 (83.3)	6
Belgium	12 (42.9)	16 (57.1)	28
Cluster 3			
Australia	26 (56.5)	20 (43.5)	46
Belgium	11 (44.0)	14 (56.0)	25

Each of the three main clusters and the majority of the subclusters contained closely related G1P[8] strains from both Belgium and Australia collected before and after rotavirus vaccine introduction (fig. 2*B*). Subclusters were identified that contain unique strains detected before (shaded green) or after vaccine introduction (shaded red), although in most cases these clusters also coincided with a single sampling location and period (fig. 2*B*). The majority of subclusters containing only strains identified after vaccine introduction were observed in cluster 3, and generally represented unique strains in either Belgium or Australia.

**Fig. 3.—:**
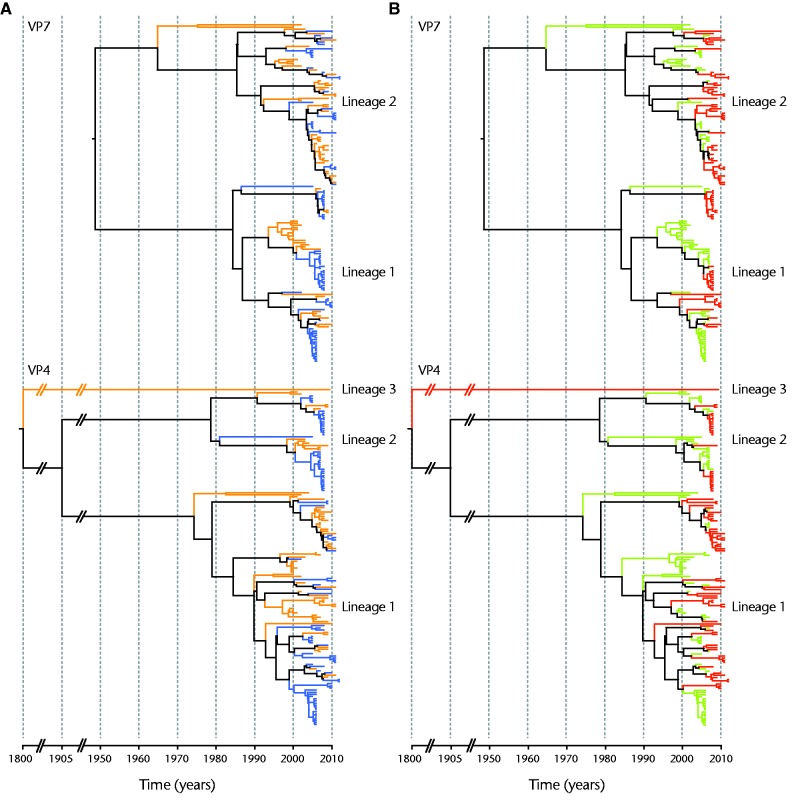
Bayesian maximum clade credibility tree based on the nucleotide sequence of 157 VP7 and VP4 gene segments. The color coding of the branches is based on the country of origin (orange for Belgium and blue for Australia) (*A*). The color coding is based on the year of isolation (green for strains isolated before rotavirus vaccine introduction and red for strains isolated after rotavirus vaccine introduction) (*B*).

To further investigate the evolutionary dynamics for the individual gene segments, we constructed phylogenetic trees for all 11 gene segments using the Bayesian phylogenetic inference of time-measures trees as implemented in the BEAST package. The VP7 and VP4 genes were segregated in two and three lineages, respectively (fig. 3). For VP7, both lineages were approximately of equal size and contained Belgian and Australian strains. Belgian and Australian strains were more intermingled in lineage 2, while separate clusters of Belgian or Australian strains were more frequently observed in lineage 1, indicating more localized epidemics in each country (fig. 3*A*). For VP4, three lineages were observed, but the majority of the strains were found in lineage 1. Belgian and Australian G1P[8] strains were more intermingled within lineage 1 compared with lineage 2 or 3. Most strains that formed closely related clusters for VP7 were also closely related for VP4, although some of these clusters show evidence of reassortment as there is no complete congruency between VP7 and VP4 lineages. Within lineage 1 and 2 several clades containing Australian G1P[8] strains were detected. One Belgian strain was distinct from all other P[8] VP4 genes characterized in this study. It clustered separately from all other Belgian and Australian G1P[8] strains and was most closely related to OP354-like P[8] VP4 genes (data not shown).

The phylogenetic trees of the other nine gene segments of Belgian and Australian G1P[8] strains also revealed the occurrence of two or more lineages for each gene segment (supplementary fig. S1, Supplementary Material online). Four gene segments possessed only two lineages (VP1, VP3, NSP1, and NSP5), while VP6, NSP3, and NSP4 possessed a third lineage comprising one or two Australian (VP6 and NSP3) or Belgian (NSP4) G1P[8] strains. The phylogenetic trees of NSP2 and VP2 revealed four and five lineages, respectively. In general, Belgian and Australian G1P[8] strains were present in all major lineages for all these gene segments, although localized clusters of closely related strains were more prominently present for the Australian strains compared with the Belgian G1P[8] strains (supplementary fig. S1, Supplementary Material online).

We also investigated the clustering patterns of Belgian and Australian G1P[8] strains with respect to their time of isolation (before or after vaccine introduction). For VP7 and VP4, G1P[8] strains isolated before and after vaccine introduction were present in the two major lineages (fig. 3*B*). However, for VP7, 60% (45 of 75) of all lineage 2 strains were isolated after vaccine introduction, whereas for lineage 1 only 44% (36 of 82) of the strains were isolated after vaccine introduction (*P* = 0.06) (fig. 2). For VP4, strains isolated before and after vaccine introduction were evenly distributed across the different lineages (51%; 24 of 47 and 56 of 109 for lineage 1 and 2, respectively; *P* = 1.00) (fig. 3*B*), but strains isolated after vaccine introduction dominated in one particular clade within lineage 1. For the other nine gene segments, strains isolated before and after vaccine introduction were also observed in all major lineages and strains isolated before and after vaccine introduction were approximately evenly distributed across the different lineages. Gene segments from G1P[8] strains isolated before and after vaccine introduction were sometimes also found to be closely related, indicating a prolonged circulation of a gene variant for an extended period of time (supplementary fig. S2, Supplementary Material online). There were sublineages present in numerous trees, such as NSP1, NSP3, and NSP4, that predominantly or exclusively comprised strains collected during the postvaccine era.

**Fig. 4.—:**
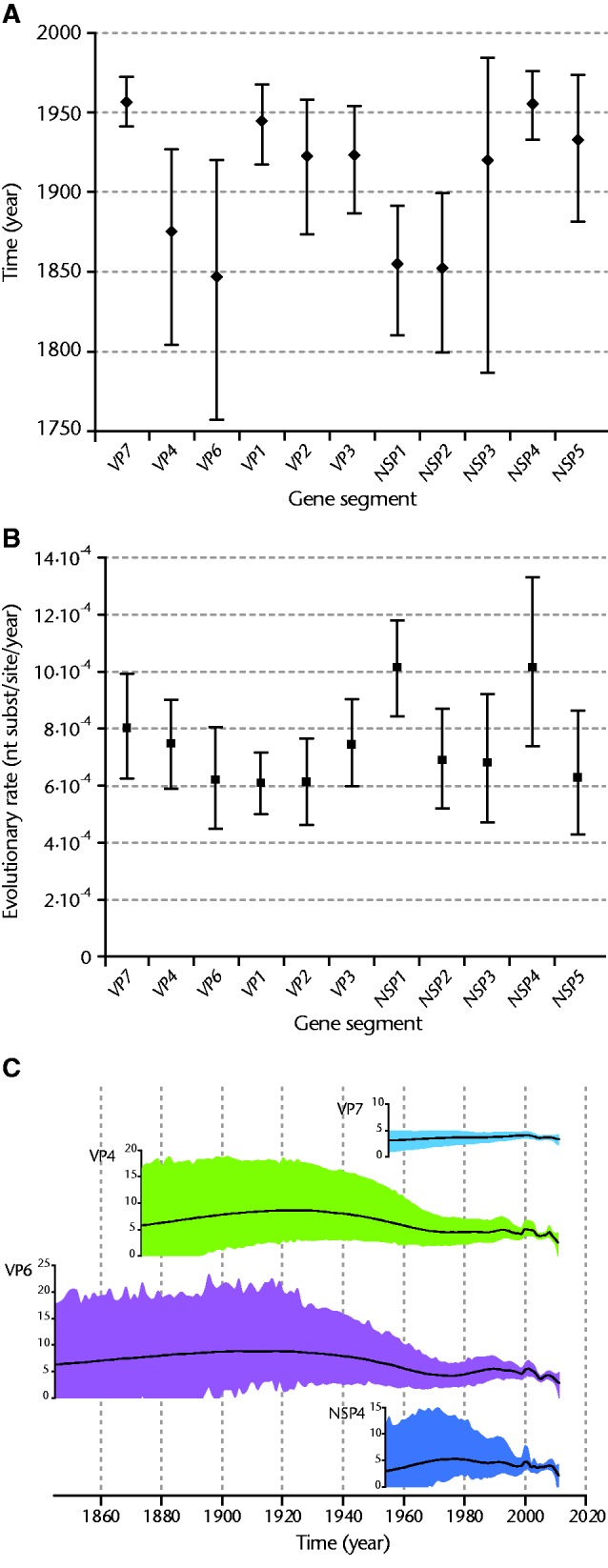
TMRCA for each gene segment based on the combined data set of Belgian and Australian G1P[8] strains. Mean TMRCAs are indicated together with their 95% HPD intervals (*A*). Evolutionary rates for each gene segment are shown together with their 95% HPD intervals (*B*). The Bayesian Skygrid plots for the *VP7*, *VP4*, *VP6*, and *NSP4* gene segments. The black line indicates the mean population size and the 95% HPD interval is indicated by the colored area around the black line (*C*).

Using the combined data set of all Belgian and Australian G1P[8] rotaviruses, we calculated the time to the most recent common ancestor (TMRCA) for all 11 gene segments. The TMRCAs ranged from 1846 to 1955 (fig. 4*A*). The youngest TMRCAs were observed for VP7 (1955; 95% highest posterior density [HPD]: 1932–1975) and NSP4 (1954; 95% HPD: 1938–1971). Other gene segments with relatively young TMRCAs were VP1, VP2, VP3, NSP3, and NSP5. Remarkably older TMRCAs were observed for the VP4, VP6, NSP1, and NSP2 gene segments. For VP4, this was partially the result of a single outlier strain in lineage 3 (the TMRCA of P[8] lineage 1 and P[8] lineage 2 strains was estimated at 1889; 95% HPD: 1832–1942), while for VP6, NSP1, and NSP2 a comparatively large genetic diversity resulted in a relatively old TMRCA (fig. 4*A*). A separate TMRCA analysis was conducted for the data sets from each country resulting in similar findings, except for VP4, NSP1, and NSP5. For these gene segments, the TMRCA of Australian strains was more recent than those of Belgian strains, due to a lower genetic diversity in the strains isolated in Australia (data not shown). We also calculated the evolutionary rate for each gene segment, which revealed only marginal differences between the different gene segments (fig. 4*B*). The evolutionary rates for each gene segment ranged from 6.05 × 10^−^^4^ to 1.01 × 10^−^^3^. The highest mutation rates were observed for NSP1 (1.01 × 10^−^^3^; 95% HPD: 8.44 × 10^−^^4^ to 1.18 × 10^−^^3^) and NSP4 (1.01 × 10^−^^3^; 95% HPD: 7.39 × 10^−^^4^ to 1.33 × 10^−^^3^), respectively.

The population size estimates through time for the Belgian and Australian VP7, VP4, VP6, and NSP4 genes showed a relative stable profile with a few peaks and troughs during the sampling period (1999–2011) (fig. 4*C*). Comparison with the Skygrid plots of other gene segments also revealed similar patterns. There is one exception, NSP1, were the peaks and troughs were absent and the Skygrid plot is completely flat. However, as the peaks and troughs were found in almost all other gene segments and were observed well before the introduction of rotavirus vaccines, they were more likely the result of sampling biases rather than vaccine introduction (supplementary fig. S3, Supplementary Material online).

Each gene segment was tested for sites under positive selection using a Belgian data set, an Australian data set, and the combined data set. Sites under positive selection were identified in VP2 (aa 40, 42), VP3 (aa 326, 683), VP7 (aa 28), and NSP1 (aa 422) by at least three of the five used models (table 2). No sites under positive selection were identified in the other seven rotavirus gene segments. Nonconservative amino acid variation was identified at the majority of sites under positive selection. However, amino acid variation at sites under positive selection did not differentiate before and after vaccine introduction in G1P[8] strains identified in either Belgian or Australian strains.

**Table 2:** Summary of Positive Selection in G1P[8] Genome Segments^a^

Genome Segment	Site	Selection Model	Amino Acid Variation
VP2			
	40 (Belgium + Australia)	MEME, SLAC, FEL, FUBAR	R/K/I
	42 (Belgium)	MEME, FEL, RC	Q/H
VP3			
	326 (Australia)	MEME, FEL, RC	K/N/E
	683 (Belgium)	MEME, FEL, RC	G/E
VP7			
	28 (Belgium + Australia)	MEME, SLAC, FEL	R/Q
NSP1			
	422 (Belgium + Australia)	MEME, FEL, RC	Q/H/K

RC, Renaissance counting.

^a^Only sites are considered that are under positive selection according to three or more out of five models (MEME, SLAC, FEL, FUBAR, or RC).

## Discussion

In this study, we investigated the impact of rotavirus vaccine introduction on the most common human rotavirus genotype G1P[8]. To our knowledge, this is the first large-scale genomic analysis of human rotaviruses collected before and after rotavirus vaccine introduction. Australia and Belgium were among the first countries worldwide where rotavirus vaccines were implemented in national immunization programs. In Belgium, the most commonly used vaccine is Rotarix, while in Australia different states are using different rotavirus vaccines (Zeller et al. 2010; Buttery et al. 2011). The samples in this study were collected from Victoria where RotaTeq is the only vaccine used (Buttery et al. 2011). Vaccination coverage was high in both countries, which provides a unique opportunity to investigate the early effect of vaccine introduction on the genetic diversity of G1P[8] rotaviruses (Braeckman et al. 2014; Hull et al. 2014). Three different clusters could be identified in the phylogenetic network that was constructed using sequences derived from the concatenation of the 11 rotavirus genes (fig. 2). Of particular interest is cluster 1, which is predominantly composed of Belgian G1P[8] strains from the prevaccine era. In Australia, no difference was found in the prevalence of G1P[8] strains in cluster 1 before and after vaccine introduction, which could possibly be the result of the different vaccines used in the national immunization programs of both countries. On the contrary, Australian G1P[8] strains belonging to cluster 2 were found more frequently after vaccine introduction, although this difference was not statistically significant as only 6.8% of all Australian G1P[8] strains were found in cluster 2. Lineage replacement has been suggested as an important evolutionary mechanism for rotaviruses to adapt to different immunological environments (McDonald et al. 2009; Zeller et al. 2012; Dennis et al. 2014; Zhang et al. 2014; Magagula et al. 2015) and the observed changes in lineage frequency could be an adaptation of the rotavirus population to evade immunological pressures of widespread vaccine use. However, when interpreting these data the different sample selection methods in Belgium and Australia should be kept in mind. In Belgium, samples were selected based on genetic diversity of VP7, whereas in Australia a random sample collection occurred. This may, for example, explain why Australian G1P[8] strains more frequently formed closely related subclusters in many gene segments as compared with Belgian strains.

For every gene segment at least two lineages were present within the Australian and Belgian G1P[8] strains, this is similar to that observed in the few other large-scale genomics studies investigating Wa-like rotaviruses (McDonald et al. 2009, 2012; Roy et al. 2014; Zhang et al. 2014; da Silva et al. 2015). No data are currently available about rotavirus vaccine effectiveness against intragenotypic lineages, but it seems unlikely that the difference in vaccine effectiveness is larger between different intragenotypic lineages than between different genotypes. Vaccine efficacy against rotavirus diarrhea of any severity for different rotavirus genotypes has been well studied and ranges between 61% and 88% for Rotarix and 88% and 95% for RotaTeq depending on the genotype (Soares-Weiser et al. 2012). In a Belgian case–control study, the vaccine effectiveness of Rotarix against the most common human genotypes ranged from 85% to 95% with only small differences between the vaccine effectiveness against Wa-like genotype strains G1, G3, and G4 (Braeckman et al. 2012; Matthijnssens et al. 2014). The differences in vaccine effectiveness between different lineages of G1P[8] strains are expected to be low. This was also suggested by our findings as only subtle differences were detected in the genetic diversity of the different rotavirus gene segments before and after vaccine introduction. For example, the majority of strains clustering in VP7 lineage 2 were isolated after vaccine introduction, whereas for VP7 lineage 1 most strains were isolated before vaccine introduction. Despite changes in genetic diversity, the rotavirus population size remained stable throughout the sampling period. As only relatively few countries were using rotavirus vaccines between 2006 and 2010, a potential effect on the rotavirus population size in Belgium and Australia could have been diluted by rotavirus strains migrating from other countries without a universal rotavirus vaccination program.

Belgium and Australia are located on the northern and southern hemisphere, respectively, and therefore experience rotavirus seasons that do not overlap each other. Despite this, we found Belgian and Australian G1P[8] rotaviruses in subsequent rotavirus seasons that were very closely related. It is unknown whether this is the result of direct transmission between Belgium and Australia or that both viruses originate from a third country, for example, in the tropics where rotavirus infections occur year round (Levy et al. 2009). However, it is likely that G1P[8] strains detected in Belgian and Australia are part of a global pool of circulating G1P[8] strains. The phylogenetic analysis revealed several lineages that circulated in the pre- and postvaccine era. Several unique genetic clusters, based on whole genome concatenation or specific gene segments that represented unique subclusters/lineages, were only present in the postvaccine introduction samples and warrant further monitoring. More extensive sampling of larger genomic data sets collected over a longer time period in Belgium and Australia as well as samples collected in different locations in the world would help to elucidate global patterns of rotavirus transmission.

Surprisingly, we found large variations in the TMRCA for different rotavirus gene segments, which could not be attributed to differences in evolutionary rates among segments (fig. 4). Especially, for VP4, VP6, NSP1, and NSP2 the TMRCAs were approximately 100 years older than those of many other gene segments and displayed a large genetic diversity (fig. 4 and supplementary fig. S1, Supplementary Material online). In contrast, the VP7 and NSP4 gene segment histories have a relatively recent TMRCA. Human rotaviruses are subdivided into Wa-like and DS-1-like genotype constellations. For most gene segments generally only two genotypes (1 or 2) are frequently present in human rotaviruses, while for VP7 six genotypes are regularly found: G1–G4, G9, and G12 (Matthijnssens and Van Ranst 2012). The conserved Wa-like genetic backbone allows for frequent reassortment and circulates in combination with varying G genotypes. Therefore, each G genotype is more limited in circulation compared with genotypes of other genes, which results in a decreased ability of G1 genotypes to accumulate mutations. For this study, only rotaviruses bearing the G1 genotype were selected and a large part of VP7 genetic diversity was thus omitted a priori. The TMRCA for the NSP4 segment was estimated at 1954, which together with a high mutation rate and limited genetic diversity suggest that the NSP4 E1 genotypes may have gone through a genetic bottleneck. The wide array of functions that have been assigned to the NSP4 protein, in particular the interaction with other VPs and its function as an enterotoxin (Hu et al. 2012), probably exercises a strong purifying selection pressure.

Selection pressure analysis of Australian and Belgian G1P[8] rotavirus strains identified several sites under positive selection in VP2, VP3, VP7, and NSP1, several of which were found in regions containing known cytotoxic T lymphocyte epitopes (VP2, VP3, and VP7), or protein domains that interact with innate immune factors (NSP1) (Franco et al. 1993, 1994; Buesa et al. 1999; Newell et al. 2013). The site under positive selection in NSP1 (aa 422) is located in the highly variable C-terminal region, involved in downregulation of the innate immune response by binding interferon-regulatory factors (IRF3, IRF5, and IRF7) and inhibiting NFκB through degradation of β-TrCP (Barro and Patton 2005; Arnold and Patton 2011). These data suggest that sequence variation at sites under positive selection in multiple VPs may be involved in immune escape of circulating G1P[8] strains. However, given the extent of genetic diversity between different G1P[8] lineages, a change in G1P[8] lineage frequency is likely to be a more dominant evolutionary mechanism to respond to selection pressures such as the introduction of rotavirus vaccines.

To conclude, we found limited differences between the Belgian and Australian G1P[8] rotaviruses, even though different vaccines were used in both locations. Despite the fact that rotavirus vaccine introduction has decreased rotavirus hospitalizations in many countries, our data suggest that rotavirus vaccination may impact the evolution of G1P[8] rotaviruses even though one of the vaccines, Rotarix, consists of a G1P[8] rotavirus strain. However, in this study, we only looked at limited rotavirus seasons after vaccine introduction in a period where only a small number of countries had implemented universal rotavirus vaccination programs, and as still little is known about the long-term evolution of rotaviruses, we cannot rule out that our results are limited by this. Therefore, an extended surveillance of G1P[8] rotaviruses would be valuable to ascertain long-term effects of vaccine introduction.

## Supplementary Material

Supplementary figures S1–S3 and table S1 are available at *Genome Biology and Evolution* online (http://www.gbe.oxfordjournals.org/).

Supplementary Data
