# Supplementary material for: Genome-Wide Evolutionary Analyses of G1P[8] Strains Isolated Before and After Rotavirus Vaccine Introduction
Source: Genome Biol Evol. 2015 Aug 8;7(9):2473–83. doi: 10.1093/gbe/evv157 (PMC4607516; doi:10.1093/gbe/evv157)
Supplement: Supplementary Data [file supp_7_9_2473__index.html]

Genome-Wide Evolutionary Analyses of G1P[8] Strains Isolated Before and After Rotavirus Vaccine Introduction — Supplementary Data 

# Genome-Wide Evolutionary Analyses of G1P[8] Strains Isolated Before and After Rotavirus Vaccine Introduction

## Supplementary Data

files

- Supplementary Data - pdf file
