# Supplementary material for: Genome-Wide Evolutionary Analyses of G1P[8] Strains Isolated Before and After Rotavirus Vaccine Introduction
Source: Genome Biol Evol. 2015 Aug 8;7(9):2473–83. doi: 10.1093/gbe/evv157 (PMC4607516; doi:10.1093/gbe/evv157)
Supplement: Supplementary Data [file supp_evv157_Supplementary_information.pdf]

Supplementary information

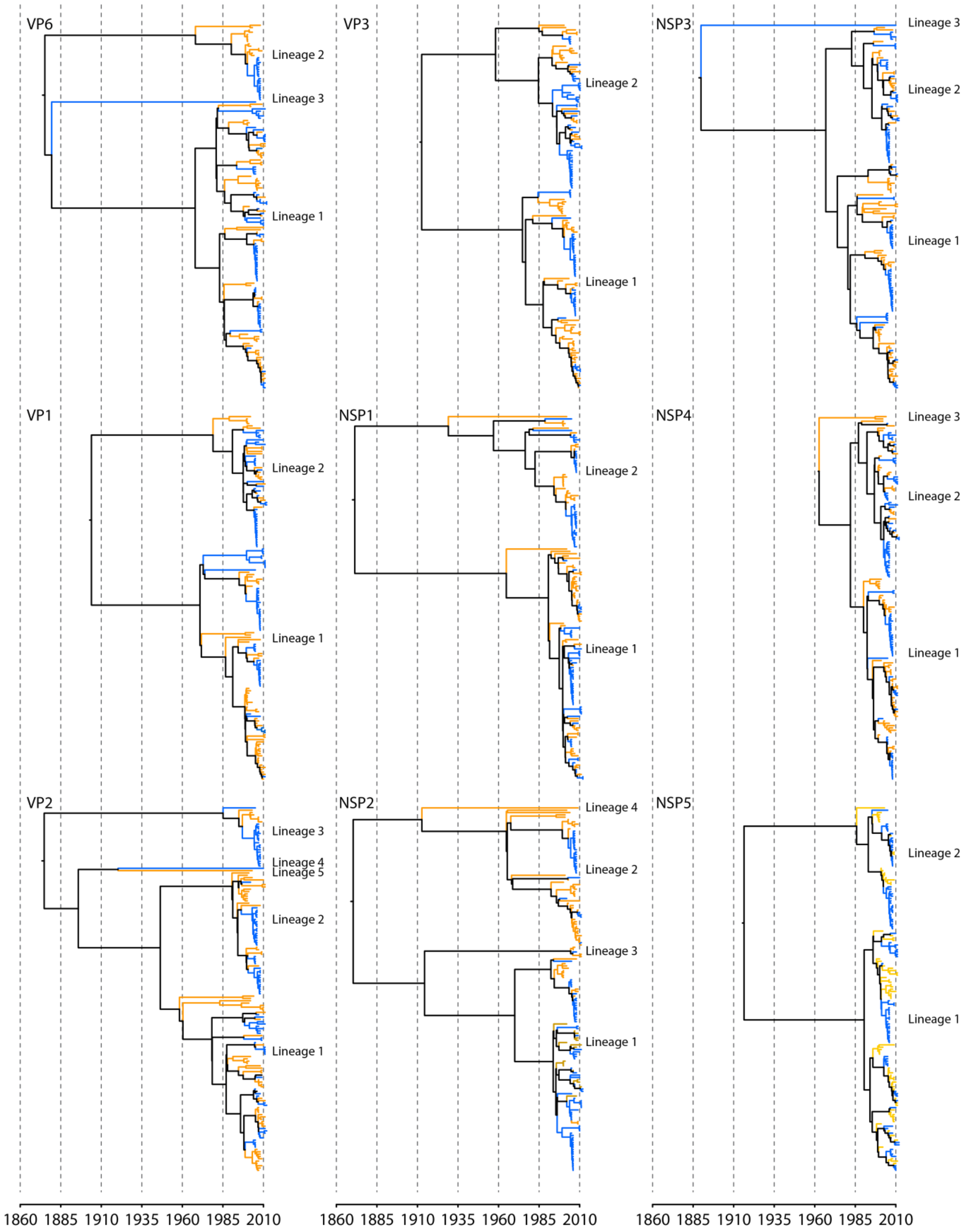

Figure S1. Bayesian maximum clade credibility trees based on the nucleotide sequence of 157 Belgian and Australian VP1-VP3, VP6 and NSP1-NSP5 gene segments. The color-coding of the branches is based on the country of origin (orange for Belgium and blue for Australia).

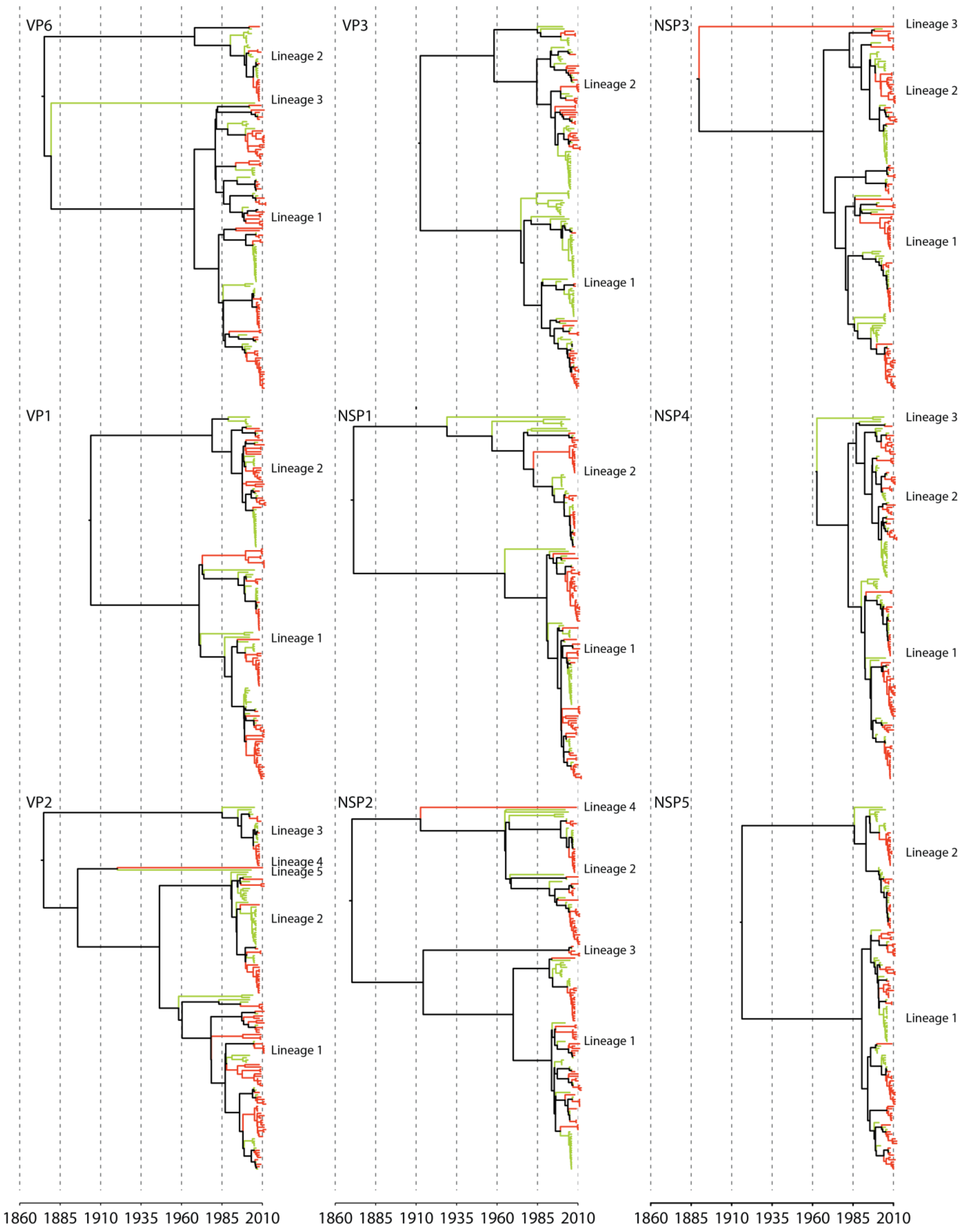

Figure S2. Bayesian maximum clade credibility trees based on the nucleotide sequence of 157 Belgian and Australian VP1-VP3, VP6 and NSP1-NSP5 gene segments. The color-coding is based on the year of isolation (green for strains isolated before rotavirus vaccine introduction and red for strains isolated after rotavirus vaccine introduction).

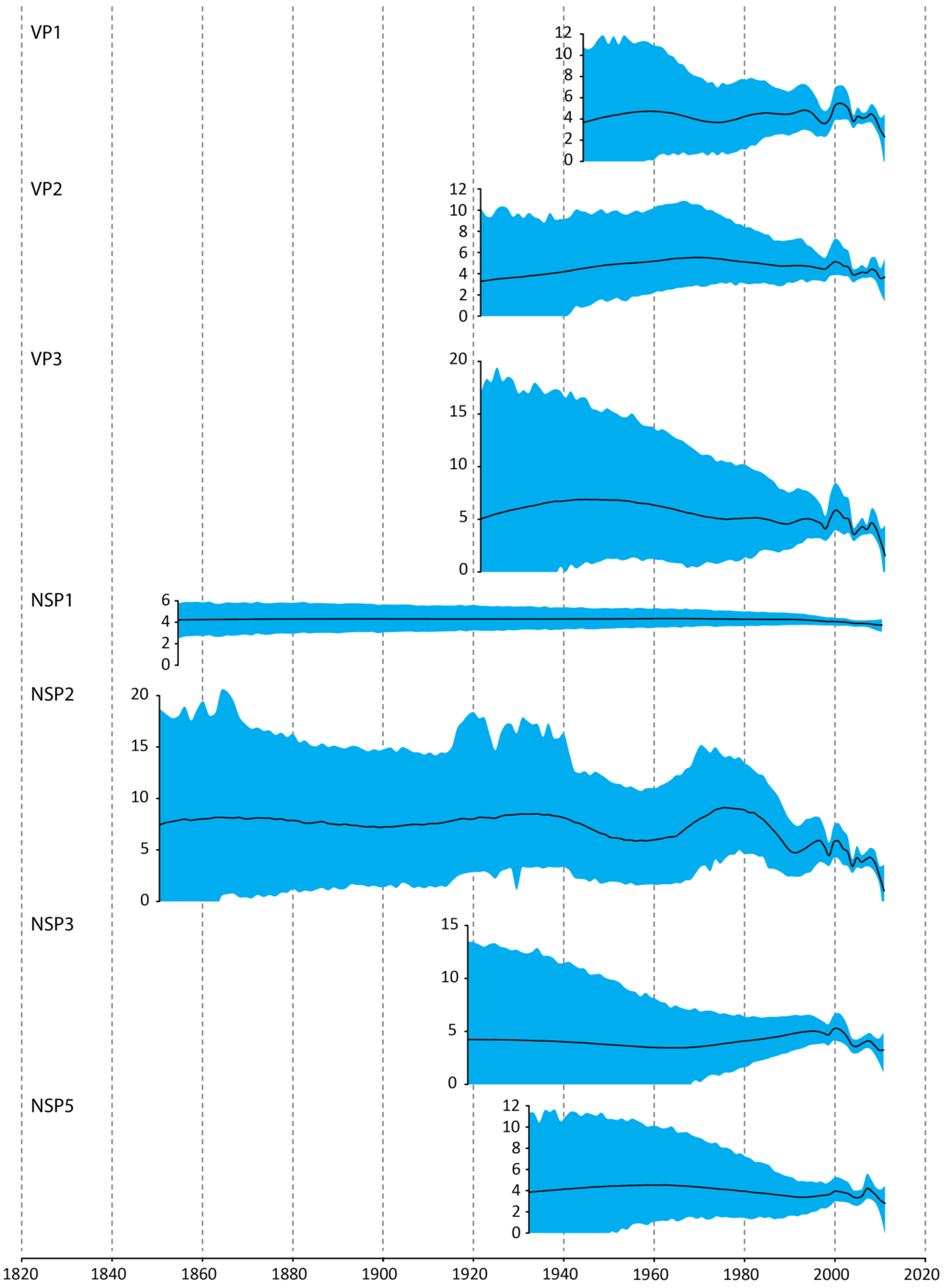

Figure S3. Bayesian skygrid plots for the VP1-VP3, NSP1-NSP3 and NSP5 gene segments. The black line indicates the mean population size and the 95% HPD interval is indicated by the blue area around the black line.

Table S1. Overview of Australian and Belgian G1P[8] strains used in this study and accession numbers for each gene segment.

	VP7	VP4	VP6	VP1	VP2	VP3	NSP1	NSP2	NSP3	NSP4	NSP5
RVA/Human-wt/AUS/CK00001/2004/G1P8	JF490088	JF490091	JF490090	JF490083	JF490093	JF490092	JF490085	JF490084	JF490089	JF490087	JF490086
RVA/Human-wt/AUS/CK00002/2004/G1P8	JF490098	JF490102	JF490100	JF490101	JF490104	JF490103	JF490095	JF490094	JF490099	JF490097	JF490096
RVA/Human-wt/AUS/CK00003/2004/G1P8	JF490110	JF490113	JF490112	JF490105	JF490115	JF490114	JF490107	JF490106	JF490111	JF490109	JF490108
RVA/Human-wt/AUS/CK00004/2004/G1P8	JF490121	JF490124	JF490123	JF490116	JF490126	JF490125	JF490118	JF490117	JF490122	JF490120	JF490119
RVA/Human-wt/AUS/CK00005/2004/G1P8	JF490135	JF490133	JF490136	JF490127	JF490134	JF490137	JF490129	JF490128	JF490132	JF490131	JF490130
RVA/Human-wt/AUS/CK00006/2004/G1P8	JF490143	JF490146	JF490145	JF490138	JF490148	JF490147	JF490140	JF490139	JF490144	JF490142	JF490141
RVA/Human-wt/AUS/CK00007/2004/G1P8	JF490154	JF490157	JF490156	JF490149	JF490159	JF490158	JF490151	JF490150	JF490155	JF490153	JF490152
RVA/Human-wt/AUS/CK00008/2004/G1P8	JF490170	JF490167	JF490166	JF490160	JF490169	JF490168	JF490162	JF490161	JF490165	JF490164	JF490163
RVA/Human-wt/AUS/CK00009/2004/G1P8	JF490181	JF490178	JF490177	JF490171	JF490180	JF490179	JF490173	JF490172	JF490176	JF490175	JF490174
RVA/Human-wt/AUS/CK00012/2004/G1P8	JF490198	JF490201	JF490200	JF490193	JF490203	JF490202	JF490195	JF490194	JF490199	JF490197	JF490196
RVA/Human-wt/AUS/CK00015/2005/G1P8	JF490220	JF490223	JF490222	JF490215	JF490225	JF490224	JF490217	JF490216	JF490221	JF490219	JF490218
RVA/Human-wt/AUS/CK00016/2005/G1P8	JF490231	JF490234	JF490233	JF490226	JF490236	JF490235	JF490228	JF490227	JF490232	JF490230	JF490229
RVA/Human-wt/AUS/CK00017/2005/G1P8	JF490242	JF490245	JF490244	JF490237	JF490247	JF490246	JF490239	JF490238	JF490243	JF490241	JF490240
RVA/Human-wt/AUS/CK00018/2005/G1P8	JF490253	JF490256	JF490255	JF490248	JF490258	JF490257	JF490250	JF490249	JF490254	JF490252	JF490251
RVA/Human-wt/AUS/CK00019/2005/G1P8	JF490263	JF490266	JF490265	JF490268	JF490269	JF490267	JF490260	JF490259	JF490264	JF490262	JF490261
RVA/Human-wt/AUS/CK00020/2005/G1P8	JF490275	JF490278	JF490277	JF490270	JF490280	JF490279	JF490272	JF490271	JF490276	JF490274	JF490273
RVA/Human-wt/AUS/CK00021/2005/G1P8	JF490286	JF490291	JF490288	JF490281	JF490290	JF490289	JF490283	JF490282	JF490287	JF490285	JF490284
RVA/Human-wt/AUS/CK00022/2005/G1P8	JF490297	JF490300	JF490299	JF490292	JF490302	JF490301	JF490294	JF490293	JF490298	JF490296	JF490295
RVA/Human-wt/AUS/CK00023/2005/G1P8	JF490308	JF490311	JF490310	JF490303	JF490313	JF490312	JF490305	JF490304	JF490309	JF490307	JF490306
RVA/Human-wt/AUS/CK00024/2005/G1P8	JF490317	JF490322	JF490319	JF490320	JF490324	JF490323	JF490321	JF490314	JF490318	JF490316	JF490315
RVA/Human-wt/AUS/CK00026/2005/G1P8	JF490330	JF490333	JF490332	JF490325	JF490335	JF490334	JF490327	JF490326	JF490331	JF490329	JF490328
RVA/Human-wt/AUS/CK00027/2005/G1P8	JF490341	JF490344	JF490343	JF490336	JF490345	JF490346	JF490338	JF490337	JF490342	JF490340	JF490339
RVA/Human-wt/AUS/CK00028/2005/G1P8	JF490351	JF490354	JF490353	JF490347	JF490356	JF490355	JF490357	JF490348	JF490352	JF490350	JF490349
RVA/Human-wt/AUS/CK00029/2006/G1P8	JF490368	JF490365	JF490364	JF490358	JF490367	JF490366	JF490360	JF490359	JF490363	JF490362	JF490361
RVA/Human-wt/AUS/CK00030/2006/G1P8	JF490374	JF490378	JF490376	JF490369	JF490379	JF490377	JF490371	JF490370	JF490375	JF490373	JF490372
RVA/Human-wt/AUS/CK00032/2006/G1P8	JF490385	JF490388	JF490387	JF490380	JF490390	JF490389	JF490382	JF490381	JF490386	JF490384	JF490383
RVA/Human-wt/AUS/CK00033/2007/G1P8	JF490396	JF490399	JF490398	JF490391	JF490401	JF490400	JF490393	JF490392	JF490397	JF490395	JF490394
RVA/Human-wt/AUS/CK00034/2007/G1P8	JF490406	JF490409	JF490408	JF490402	JF490412	JF490411	JF490410	JF490403	JF490407	JF490405	JF490404
RVA/Human-wt/AUS/CK00035/2005/G1P8	JF490418	JF490421	JF490420	JF490413	JF490423	JF490422	JF490415	JF490414	JF490419	JF490417	JF490416
RVA/Human-wt/AUS/CK00036/2005/G1P8	JF490428	JF490431	JF490430	JF490424	JF490433	JF490432	JF490434	JF490425	JF490429	JF490427	JF490426
RVA/Human-wt/AUS/CK00037/2006/G1P8	JF490444	JF490441	JF490440	JF490435	JF490445	JF490442	JF490443	JF490436	JF490439	JF490438	JF490437
RVA/Human-wt/AUS/CK00039/2006/G1P8	JF490451	JF490454	JF490453	JF490446	JF490456	JF490455	JF490448	JF490447	JF490452	JF490450	JF490449
RVA/Human-wt/AUS/CK00040/2006/G1P8	JF490462	JF490465	JF490464	JF490457	JF490467	JF490466	JF490459	JF490458	JF490463	JF490461	JF490460
RVA/Human-wt/AUS/CK00041/2006/G1P8	JF490473	JF490476	JF490475	JF490468	JF490478	JF490477	JF490470	JF490469	JF490474	JF490472	JF490471
RVA/Human-wt/AUS/CK00043/2006/G1P8	JF490484	JF490487	JF490486	JF490479	JF490489	JF490488	JF490481	JF490480	JF490485	JF490483	JF490482
RVA/Human-wt/AUS/CK00045/2006/G1P8	JF490498	JF490491	JF490500	JF490490	JF490493	JF490492	JF490495	JF490494	JF490499	JF490497	JF490496
RVA/Human-wt/AUS/CK00046/2006/G1P8	JF490506	JF490509	JF490508	JF490501	JF490511	JF490510	JF490503	JF490502	JF490507	JF490505	JF490504
RVA/Human-wt/AUS/CK00047/2006/G1P8	JF490517	JF490520	JF490519	JF490512	JF490522	JF490521	JF490514	JF490513	JF490518	JF490516	JF490515
RVA/Human-wt/AUS/CK00048/2004/G1P8	JF490528	JF490531	JF490530	JF490523	JF490533	JF490532	JF490525	JF490524	JF490529	JF490527	JF490526
RVA/Human-wt/AUS/CK00049/2004/G1P8	JF490539	JF490543	JF490541	JF490534	JF490544	JF490542	JF490536	JF490535	JF490540	JF490538	JF490537
RVA/Human-wt/AUS/CK00050/2005/G1P8	JF490549	JF490554	JF490551	JF490545	JF490555	JF490552	JF490553	JF490546	JF490550	JF490548	JF490547
RVA/Human-wt/AUS/CK00051/2007/G1P8	JX027645	JX027640	JX027642	JX027637	JX027638	JX027639	JX027641	JX027644	JX027643	JX027646	JX027647
RVA/Human-wt/AUS/CK00052/2007/G1P8	JX027656	JX027651	JX027653	JX027648	JX027649	JX027650	JX027652	JX027655	JX027654	JX027657	JX027658
RVA/Human-wt/AUS/CK00053/2007/G1P8	JX027667	JX027662	JX027664	JX027659	JX027660	JX027661	JX027663	JX027666	JX027665	JX027668	JX027669
RVA/Human-wt/AUS/CK00054/2007/G1P8	JX027678	JX027673	JX027675	JX027670	JX027671	JX027672	JX027674	JX027677	JX027676	JX027679	JX027680
RVA/Human-wt/AUS/CK00056/2007/G1P8	JX027689	JX027684	JX027686	JX027681	JX027682	JX027683	JX027685	JX027688	JX027687	JX027690	JX027691
RVA/Human-wt/AUS/CK00057/2007/G1P8	KC769321	KC769319	KC769320	KC769316	KC769317	KC769318	KC769311	KC769313	KC769312	KC769314	KC769315
RVA/Human-wt/AUS/CK00058/2007/G1P8	KC769332	KC769330	KC769331	KC769327	KC769328	KC769329	KC769322	KC769323	KC769324	KC769325	KC769326

RVA/Human-wt/AUS/CK00060/2007/G1P8	JX027699	JX027702	JX027696	JX027692	JX027693	JX027694	JX027695	JX027698	JX027697	JX027700	JX027701
RVA/Human-wt/AUS/CK00062/2007/G1P8	JX027709	JX027707	JX027706	JX027708	JX027712	JX027711	JX027713	JX027703	JX027710	JX027705	JX027704
RVA/Human-wt/AUS/CK00063/2007/G1P8	JX027718	JX027722	JX027720	JX027714	JX027724	JX027723	JX027721	JX027715	JX027719	JX027717	JX027716
RVA/Human-wt/AUS/CK00067/2007/G1P8	JX027729	JX027733	JX027731	JX027732	JX027735	JX027734	JX027726	JX027725	JX027730	JX027728	JX027727
RVA/Human-wt/AUS/CK00069/2007/G1P8	KC769409	KC769407	KC769408	KC769404	KC769405	KC769406	KC769399	KC769400	KC769401	KC769402	KC769403
RVA/Human-wt/AUS/CK00070/2007/G1P8	KC769420	KC769418	KC769419	KC769415	KC769416	KC769417	KC769410	KC769411	KC769412	KC769413	KC769414
RVA/Human-wt/AUS/CK00071/2007/G1P8	KC769431	KC769429	KC769430	KC769426	KC769427	KC769428	KC769421	KC769422	KC769423	KC769424	KC769425
RVA/Human-wt/AUS/CK00072/2007/G1P8	JX027741	JX027744	JX027743	JX027736	JX027745	JX027746	JX027738	JX027737	JX027742	JX027740	JX027739
RVA/Human-wt/AUS/CK00073/2007/G1P8	KC769442	KC769440	KC769441	KC769437	KC769438	KC769439	KC769432	KC769433	KC769434	KC769435	KC769436
RVA/Human-wt/AUS/CK00074/2007/G1P8	JX027751	JX027755	JX027753	JX027747	JX027754	JX027757	JX027756	JX027748	JX027752	JX027750	JX027749
RVA/Human-wt/AUS/CK00075/2007/G1P8	JX027763	JX027766	JX027765	JX027758	JX027768	JX027767	JX027760	JX027759	JX027764	JX027762	JX027761
RVA/Human-wt/AUS/CK00076/2007/G1P8	JX027773	JX027779	JX027775	JX027778	JX027777	JX027776	JX027770	JX027769	JX027774	JX027772	JX027771
RVA/Human-wt/AUS/CK00077/2007/G1P8	JX027785	JX027789	JX027787	JX027780	JX027788	JX027790	JX027782	JX027781	JX027786	JX027784	JX027783
RVA/Human-wt/AUS/CK00078/2007/G1P8	JX027796	JX027799	JX027798	JX027791	JX027801	JX027800	JX027793	JX027792	JX027797	JX027795	JX027794
RVA/Human-wt/AUS/CK00079/2007/G1P8	KC769453	KC769451	KC769452	KC769448	KC769449	KC769450	KC769443	KC769444	KC769445	KC769446	KC769447
RVA/Human-wt/AUS/CK00080/2007/G1P8	KC769464	KC769462	KC769463	KC769459	KC769460	KC769461	KC769454	KC769455	KC769456	KC769457	KC769458
RVA/Human-wt/AUS/CK00081/2007/G1P8	KC195774	KC195772	KC195773	KC195769	KC195770	KC195771	KC195764	KC195765	KC195766	KC195767	KC195768
RVA/Human-wt/AUS/CK00082/2007/G1P8	JX027806	JX027812	JX027808	JX027802	JX027810	JX027809	JX027811	JX027803	JX027807	JX027805	JX027804
RVA/Human-wt/AUS/CK00083/2008/G1P8	JX027818	JX027821	JX027820	JX027813	JX027823	JX027822	JX027815	JX027814	JX027819	JX027817	JX027816
RVA/Human-wt/AUS/CK00084/2008/G1P8	JX027828	JX027831	JX027830	JX027824	JX027834	JX027833	JX027832	JX027825	JX027829	JX027827	JX027826
RVA/Human-wt/AUS/CK00085/2008/G1P8	JX027840	JX027843	JX027842	JX027835	JX027845	JX027844	JX027837	JX027836	JX027841	JX027839	JX027838
RVA/Human-wt/AUS/CK00086/2009/G1P8	JX027850	JX027853	JX027852	JX027856	JX027855	JX027854	JX027847	JX027846	JX027851	JX027849	JX027848
RVA/Human-wt/AUS/CK00087/2009/G1P8	JX027864	JX027865	JX027862	JX027857	JX027863	JX027867	JX027866	JX027858	JX027861	JX027860	JX027859
RVA/Human-wt/AUS/CK00088/2009/G1P8	JX027873	JX027876	JX027875	JX027868	JX027878	JX027877	JX027870	JX027869	JX027874	JX027872	JX027871
RVA/Human-wt/AUS/CK00089/2009/G1P8	JX027884	JX027887	JX027886	JX027879	JX027889	JX027888	JX027881	JX027880	JX027885	JX027883	JX027882
RVA/Human-wt/AUS/CK00091/2009/G1P8	JX027895	JX027898	JX027897	JX027890	JX027900	JX027899	JX027892	JX027891	JX027896	JX027894	JX027893
RVA/Human-wt/AUS/CK00092/2009/G1P8	JX027905	JX027911	JX027907	JX027908	JX027909	JX027910	JX027902	JX027901	JX027906	JX027904	JX027903
RVA/Human-wt/AUS/CK00093/2009/G1P8	KC195763	KC195759	KC195758	KC195753	KC195761	KC195760	KC195762	KC195754	KC195757	KC195756	KC195755
RVA/Human-wt/AUS/CK00094/2009/G1P8	JX027917	JX027920	JX027918	JX027912	JX027921	JX027922	JX027914	JX027913	JX027919	JX027916	JX027915
RVA/Human-wt/AUS/CK00095/2010/G1P8	JX027927	JX027933	JX027929	JX027930	JX027931	JX027932	JX027924	JX027923	JX027928	JX027926	JX027925
RVA/Human-wt/AUS/CK00101/2010/G1P8	KP645261	KP645259	KP645260	KP645256	KP645257	KP645258	KP645262	KP645263	KP645264	KP645265	KP645266
RVA/Human-wt/AUS/CK00102/2010/G1P8	KP645272	KP645270	KP645271	KP645267	KP645268	KP645269	KP645273	KP645274	KP645275	KP645276	KP645277
RVA/Human-wt/AUS/CK00103/2010/G1P8	KP645283	KP645281	KP645282	KP645278	KP645279	KP645280	KP645284	KP645285	KP645286	KP645287	KP645288
RVA/Human-wt/AUS/CK00104/2010/G1P8	KP645294	KP645292	KP645293	KP645289	KP645290	KP645291	KP645295	KP645296	KP645297	KP645298	KP645299
RVA/Human-wt/AUS/CK00105/2010/G1P8	KP645305	KP645303	KP645304	KP645300	KP645301	KP645302	KP645306	KP645307	KP645308	KP645309	KP645310
RVA/Human-wt/AUS/CK00106/2010/G1P8	KP645316	KP645314	KP645315	KP645311	KP645312	KP645313	KP645317	KP645318	KP645319	KP645320	KP645321
RVA/Human-wt/AUS/CK00108/2011/G1P8	KP645327	KP645325	KP645326	KP645322	KP645323	KP645324	KP645328	KP645329	KP645330	KP645331	KP645332
RVA/Human-wt/AUS/CK00110/2011/G1P8	KP645338	KP645336	KP645337	KP645333	KP645334	KP645335	KP645339	KP645340	KP645341	KP645342	KP645343
RVA/Human-wt/AUS/CK20019/2001/G1P8	KC443581	KC443579	KC443580	KC443576	KC443577	KC443578	KC443571	KC443572	KC443573	KC443574	KC443575
RVA/Human-wt/AUS/CK20043/2010/G1P8	KC443493	KC443491	KC443492	KC443488	KC443489	KC443490	KC443483	KC443484	KC443485	KC443486	KC443487
RVA/Human-wt/BEL/BE00004/2005/G1P8	HQ392009	HQ392011	HQ392007	HQ392003	HQ392012	HQ392008	HQ392013	HQ392004	HQ392010	HQ392006	HQ392005
RVA/Human-wt/BEL/BE00006/2005/G1P8	HQ392022	HQ392018	HQ392017	HQ392021	HQ392020	HQ392019	HQ392024	HQ392014	HQ392023	HQ392016	HQ392015
RVA/Human-wt/BEL/BE00007/2005/G1P8	HQ392034	HQ392030	HQ392029	HQ392031	HQ392032	HQ392035	HQ392033	HQ392025	HQ392028	HQ392027	HQ392026
RVA/Human-wt/BEL/BE00009/2005/G1P8	HQ392040	HQ392042	HQ392041	HQ392044	HQ392046	HQ392043	HQ392037	HQ392036	HQ392045	HQ392039	HQ392038
RVA/Human-wt/BEL/BE00010/2006/G1P8	HQ392051	HQ392057	HQ392052	HQ392055	HQ392054	HQ392053	HQ392048	HQ392047	HQ392056	HQ392050	HQ392049
RVA/Human-wt/BEL/BE00012/2006/G1P8	HQ392058	HQ392067	HQ392059	HQ392062	HQ392066	HQ392064	HQ392063	HQ392061	HQ392060	HQ392068	HQ392065
RVA/Human-wt/BEL/BE00013/2006/G1P8	HQ392074	HQ392077	HQ392076	HQ392069	HQ392079	HQ392078	HQ392071	HQ392070	HQ392075	HQ392073	HQ392072
RVA/Human-wt/BEL/BE00014/2006/G1P8	HQ392088	HQ392084	HQ392083	HQ392086	HQ392085	HQ392090	HQ392087	HQ392080	HQ392089	HQ392082	HQ392081
RVA/Human-wt/BEL/BE00015/2006/G1P8	HQ392100	HQ392096	HQ392094	HQ392095	HQ392098	HQ392097	HQ392099	HQ392091	HQ392101	HQ392093	HQ392092
RVA/Human-wt/BEL/BE00016/2006/G1P8	HQ392111	HQ392107	HQ392106	HQ392110	HQ392109	HQ392108	HQ392103	HQ392102	HQ392112	HQ392105	HQ392104
RVA/Human-wt/BEL/BE00017/2006/G1P8	HQ392122	HQ392119	HQ392118	HQ392113	HQ392121	HQ392120	HQ392115	HQ392114	HQ392123	HQ392117	HQ392116
RVA/Human-wt/BEL/BE00018/2006/G1P8	HQ392132	HQ392129	HQ392128	HQ392124	HQ392130	HQ392134	HQ392131	HQ392125	HQ392133	HQ392127	HQ392126

RVA/Human-wt/BEL/BE00019/2006/G1P8	HQ392144	HQ392140	HQ392139	HQ392135	HQ392142	HQ392141	HQ392143	HQ392136	HQ392145	HQ392138	HQ392137
RVA/Human-wt/BEL/BE00020/2006/G1P8	HQ392150	HQ392153	HQ392152	HQ392146	HQ392155	HQ392156	HQ392154	HQ392147	HQ392151	HQ392149	HQ392148
RVA/Human-wt/BEL/BE00021/2007/G1P8	HQ392167	HQ392164	HQ392161	HQ392157	HQ392162	HQ392165	HQ392166	HQ392158	HQ392163	HQ392160	HQ392159
RVA/Human-wt/BEL/BE00022/2007/G1P8	HQ392176	HQ392178	HQ392171	HQ392168	HQ392173	HQ392172	HQ392174	HQ392169	HQ392177	HQ392170	HQ392175
RVA/Human-wt/BEL/BE00023/2007/G1P8	HQ392183	HQ392185	HQ392184	HQ392179	HQ392187	HQ392186	HQ392181	HQ392180	HQ392189	HQ392188	HQ392182
RVA/Human-wt/BEL/BE00024/2007/G1P8	HQ392198	HQ392194	HQ392200	HQ392197	HQ392196	HQ392195	HQ392191	HQ392190	HQ392199	HQ392193	HQ392192
RVA/Human-wt/BEL/BE00025/2007/G1P8	HQ392204	HQ392206	HQ392205	HQ392209	HQ392208	HQ392207	HQ392210	HQ392201	HQ392211	HQ392203	HQ392202
RVA/Human-wt/BEL/BE00027/2008/G1P8	HQ392217	HQ392219	HQ392218	HQ392212	HQ392221	HQ392220	HQ392214	HQ392213	HQ392222	HQ392216	HQ392215
RVA/Human-wt/BEL/BE00028/2007/G1P8	HQ392232	HQ392229	HQ392228	HQ392223	HQ392231	HQ392230	HQ392225	HQ392224	HQ392233	HQ392227	HQ392226
RVA/Human-wt/BEL/BE00029/2008/G1P8	HQ392244	HQ392238	HQ392237	HQ392239	HQ392242	HQ392241	HQ392243	HQ392234	HQ392240	HQ392236	HQ392235
RVA/Human-wt/BEL/BE00030/2008/G1P8	HQ392250	HQ392253	HQ392252	HQ392245	HQ392254	HQ392255	HQ392247	HQ392246	HQ392251	HQ392249	HQ392248
RVA/Human-wt/BEL/BE00031/2008/G1P8	HQ392261	HQ392263	HQ392262	HQ392256	HQ392265	HQ392264	HQ392258	HQ392257	HQ392266	HQ392260	HQ392259
RVA/Human-wt/BEL/BE00032/2008/G1P8	HQ392272	HQ392275	HQ392274	HQ392267	HQ392277	HQ392276	HQ392269	HQ392268	HQ392273	HQ392271	HQ392270
RVA/Human-wt/BEL/BE00033/2008/G1P8	HQ392283	HQ392286	HQ392285	HQ392278	HQ392288	HQ392287	HQ392280	HQ392279	HQ392284	HQ392282	HQ392281
RVA/Human-wt/BEL/BE00034/2008/G1P8	HQ392294	HQ392297	HQ392296	HQ392289	HQ392299	HQ392298	HQ392291	HQ392290	HQ392295	HQ392293	HQ392292
RVA/Human-wt/BEL/BE00035/2008/G1P8	HQ392309	HQ392306	HQ392305	HQ392300	HQ392308	HQ392307	HQ392302	HQ392301	HQ392310	HQ392304	HQ392303
RVA/Human-wt/BEL/BE00036/2008/G1P8	HQ392321	HQ392317	HQ392316	HQ392311	HQ392318	HQ392320	HQ392313	HQ392312	HQ392319	HQ392315	HQ392314
RVA/Human-wt/BEL/BE00037/2008/G1P8	HQ392326	HQ392329	HQ392327	HQ392328	HQ392331	HQ392330	HQ392323	HQ392322	HQ392332	HQ392325	HQ392324
RVA/Human-wt/BEL/BE00038/2008/G1P8	HQ392337	HQ392340	HQ392338	HQ392339	HQ392341	HQ392343	HQ392334	HQ392333	HQ392342	HQ392336	HQ392335
RVA/Human-wt/BEL/BE00039/2008/G1P8	HQ392352	HQ392354	HQ392348	HQ392349	HQ392351	HQ392350	HQ392345	HQ392344	HQ392353	HQ392347	HQ392346
RVA/Human-wt/BEL/BE00040/2008/G1P8	HQ392360	HQ392362	HQ392361	HQ392355	HQ392364	HQ392363	HQ392357	HQ392356	HQ392365	HQ392359	HQ392358
RVA/Human-wt/BEL/BE00041/2007/G1P8	HQ392431	HQ392428	HQ392427	HQ392421	HQ392430	HQ392429	HQ392423	HQ392422	HQ392426	HQ392425	HQ392424
RVA/Human-wt/BEL/BE00042/2008/G1P8	HQ392371	HQ392374	HQ392373	HQ392366	HQ392376	HQ392375	HQ392368	HQ392367	HQ392372	HQ392370	HQ392369
RVA/Human-wt/BEL/BE00043/2009/G1P8	HQ392381	HQ392384	HQ392383	HQ392377	HQ392386	HQ392385	HQ392379	HQ392378	HQ392387	HQ392382	HQ392380
RVA/Human-wt/BEL/BE00044/2009/G1P8	HQ392388	HQ392398	HQ392389	HQ392391	HQ392395	HQ392393	HQ392392	HQ392390	HQ392397	HQ392396	HQ392394
RVA/Human-wt/BEL/BE00045/2009/G1P8	HQ392406	HQ392409	HQ392404	HQ392399	HQ392405	HQ392408	HQ392401	HQ392400	HQ392407	HQ392403	HQ392402
RVA/Human-wt/BEL/BE00046/2008/G1P8	HQ392437	HQ392439	HQ392438	HQ392432	HQ392441	HQ392440	HQ392434	HQ392433	HQ392442	HQ392436	HQ392435
RVA/Human-wt/BEL/BE00047/2009/G1P8	HQ392453	HQ392449	HQ392448	HQ392443	HQ392451	HQ392450	HQ392445	HQ392444	HQ392452	HQ392447	HQ392446
RVA/Human-wt/BEL/BE00049/2009/G1P8	HQ392461	HQ392458	HQ392457	HQ392463	HQ392462	HQ392464	HQ392454	HQ392459	HQ392456	HQ392460	HQ392455
RVA/Human-wt/Bel/BE00051/1999/G1P8	KT223452	JN635535	JN635536	JN635532	KT223471	KT223462	KT223443	JN635528	JN635529	JN635530	JN635531
RVA/Human-wt/Bel/BE00052/1999/G1P8	JN258786	JN258787	JN258780	JN258783	JN258789	JN258788	JN258790	JN258782	JN258781	JN258785	JN258784
RVA/Human-wt/Bel/BE00055/1999/G1P8	JN258791	JN258798	JN258792	JN258795	JN258800	JN258799	JN258801	JN258794	JN258793	JN258797	JN258796
RVA/Human-wt/Bel/BE00056/1999/G1P8	JN651758	JN651756	JN651757	KT223479	KT223472	KT223463	KT223444	JN651749	JN651750	JN651751	JN651752
RVA/Human-wt/Bel/BE00057/1999/G1P8	JN651769	JN651767	JN651768	JN651764	KT223470	KT223460	KT223441	JN651760	JN651761	JN651762	JN651763
RVA/Human-wt/Bel/BE00058/1999/G1P8	KT223453	KT223455	JN651779	JN651775	KT223474	KT223465	KT223446	JN651771	JN651772	JN651773	JN651774
RVA/Human-wt/Bel/BE00059/1999/G1P8	JN651791	KT223458	JN651790	KT223482	KT223477	KT223468	KT223449	JN651782	JN651783	KT223437	KT223436
RVA/Human-wt/Bel/BE00061/2000/G1P8	KT248550	KC193626	KC193627	KC193623	KC193624	KC193625	KC193618	KC193619	KC193620	KC193621	KC193622
RVA/Human-wt/Bel/BE00064/2000/G1P8	JN651802	JN651800	JN651801	JN651797	JN651798	KT223459	KT223440	JN651793	JN651794	JN651795	JN651796
RVA/Human-wt/Bel/BE00066/2000/G1P8	KT223454	JN651811	JN651812	JN651808	KT223478	KT223469	KT223450	KT223439	JN651805	JN651806	JN651807
RVA/Human-wt/Bel/BE00067/2000/G1P8	JN258805	JN258809	JN258807	JN258808	JN258812	JN258811	JN258810	JN258802	JN258806	JN258804	JN258803
RVA/Human-wt/Bel/BE00068/2000/G1P8	JN258813	JN258819	JN258814	JN258817	JN258823	JN258822	JN258821	JN258816	JN258815	JN258820	JN258818
RVA/Human-wt/Bel/BE00070/2001/G1P8	JN651823	KT223456	JN651822	KT223480	KT223475	KT223466	KT223447	JN651814	JN651815	JN651816	JN651817
RVA/Human-wt/Bel/BE00073/2001/G1P8	JN258827	JN258832	JN258829	JN258830	JN258831	JN258834	JN258833	JN258824	JN258828	JN258826	JN258825
RVA/Human-wt/Bel/BE00076/2001/G1P8	JN258844	JN258841	JN258839	JN258835	JN258843	JN258842	JN258845	JN258836	JN258840	JN258838	JN258837
RVA/Human-wt/Bel/BE00079/2002/G1P8	JN258846	JN258855	JN258847	JN258853	JN258851	JN258856	JN258854	JN258849	JN258848	JN258852	JN258850
RVA/Human-wt/Bel/BE00082/2002/G1P8	JN258857	JN258866	JN258858	JN258861	JN258865	JN258863	JN258862	JN258860	JN258859	JN258867	JN258864
RVA/Human-wt/Bel/BE00085/2002/G1P8	JN258874	JN258875	JN258872	JN258868	JN258877	JN258876	JN258878	JN258873	JN258871	JN258870	JN258869
RVA/Human-wt/Bel/BE00089/2002/G1P8	JN651834	KT223457	JN651833	KT223481	KT223476	KT223467	KT223448	JN651825	JN651826	JN651827	JN651828
RVA/Human-wt/Bel/BE00090/2003/G1P8	JN258879	JN258884	JN258880	JN258886	JN258889	JN258888	JN258887	JN258882	JN258881	JN258885	JN258883
RVA/Human-wt/Bel/BE00093/2003/G1P8	JN258895	JN258898	JN258897	JN258890	JN258900	JN258899	JN258892	JN258891	JN258896	JN258894	JN258893
RVA/Human-wt/Bel/BE00094/2010/G1P8	JN258908	JN258909	JN258906	JN258901	JN258911	JN258910	JN258907	JN258902	JN258905	JN258904	JN258903
RVA/Human-wt/Bel/BE00096/2010/G1P8	JN651845	JN651843	JN651844	JN651840	KT223473	KT223464	KT223445	KT223438	JN651836	JN651838	JN651839

RVA/Human-wt/Bel/BE00097/2010/G1P8	JN258917	JN258920	JN258919	JN258912	JN258921	JN258922	JN258914	JN258913	JN258918	JN258916	JN258915
RVA/Human-wt/Bel/BE00098/2010/G1P8	JN258928	JN258933	JN258930	JN258923	JN258932	JN258931	JN258925	JN258924	JN258929	JN258927	JN258926
RVA/Human-wt/Bel/BE00100/2010/G1P8	JN258941	JN258940	JN258939	JN258934	JN258943	JN258942	JN258944	JN258935	JN258938	JN258937	JN258936
RVA/Human-wt/Bel/BE00108/2010/G1P8	KT223451	JN651887	JN651888	JN651884	JN651885	KT223461	KT223442	JN651880	JN651881	JN651882	JN651883
RVA/Human-wt/Bel/BE00112/2001/G1P8	JN258953	JN258954	JN258948	JN258950	JN258949	JN258955	JN258951	JN258945	JN258947	JN258952	JN258946
